# Wealth, income and dementia in Germany: longitudinal findings from a representative survey among the oldest old

**DOI:** 10.1186/s12889-025-24239-1

**Published:** 2025-08-29

**Authors:** André Hajek, Hans-Helmut König, Snorri Bjorn Rafnsson, Razak M. Gyasi

**Affiliations:** 1 Department of Health Economics and Health Services Research, Hamburg Center for Health Economics, University Medical Center Hamburg-Eppendorf, Hamburg, Germany; 2 The Geller Institute of Ageing and Memory, School of Medicine and Biosciences, University of West London, London, UK; 3 African Population and Health Research Center, Nairobi, Kenya; 4 National Centre for Naturopathic Medicine, Faculty of Health, Southern Cross University, Lismore, NSW Australia

**Keywords:** Aged, 80 and over, Dementia, Wealth, Asset, Income

## Abstract

**Background:**

There is limited knowledge on the link between wealth, as well as income, and dementia risk among the *oldest old* (80 years and over). The purpose of our study was to examine the association between income, wealth, and dementia among the oldest old in Germany.

**Methods:**

We used representative longitudinal data from a survey, which aimed to assess the quality of life and subjective well-being of individuals aged 80 years and above in North Rhine-Westphalia (Germany). The length of follow-up was approximately 2 years. The sample consisted of both community-dwelling and institutionalized individuals, with a total of 943 observations included in the analysis. The average age of the participants was 86.0 years (SD: 4.0 years). Probable dementia was assessed by DemTect, a widely accepted screening instrument. Common income and wealth categories were used to quantify these variables.

**Results:**

After adjusting for sociodemographic and health variables, logistic random effects regressions showed that greater wealth was associated with a lower likelihood of probable dementia (second lowest wealth quartile compared to lowest wealth quartile: OR: 0.23, 95% CI: 0.07-0.76; second highest wealth quartile: OR: 0.05, 95% CI: 0.01-0.31; highest wealth quartile: OR: 0.12, 95% CI: 0.02-0.91). In contrast, income was not significantly associated with the likelihood of probable dementia.

**Conclusion:**

Our study showed a link between wealth and dementia. Such knowledge can enrich poverty and inequality research within this field. More specifically, our findings lend support to evidence-based policies aiming to decrease the burden of dementia in later life through reduction in economic inequalities and better income distribution.

**Supplementary Information:**

The online version contains supplementary material available at 10.1186/s12889-025-24239-1.

## Introduction

In developed nations like Germany, the population is experiencing an ongoing process of aging, with a notable increase in the number of individuals reaching advanced ages, particularly those aged 80 years and above. Like many physical and psychosomatic disorders, the risk of dementia is known to increase with age [1].

Dementia is a progressive cognitive impairment syndrome, most commonly attributed to Alzheimer’s disease. Recent systematic reviews and meta-analyses have revealed that the prevalence of all-type dementia is about 15% among those aged 80 to 89 years, nearly 36% among individuals aged 90 to 99 years, and approximately 66% among those aged 100 years and above [1].

Individuals with dementia face an elevated risk of reporting, among other things, a diminished health-related quality of life and expressing feelings of isolation [2]. As the disease advances, managing daily activities becomes progressively challenging for individuals living with dementia. The advancement of the disease also impacts upon families of those with dementia, who may take on tasks such as managing financial issues, or undertaking tasks such as dressing, for the individual. Societal implications include the increased demand and cost of healthcare for this population [3].

Individuals with dementia often require extensive care and supervision [4]. This condition increases the risk of nursing home admissions and premature mortality [5]. Furthermore, dementia imposes a significant economic burden [6]. For instance, the global societal cost per year of dementia amounted to approximately US$1313 billion for 55.2 million individuals affected by dementia [6]. Given the diverse detrimental consequences of dementia, investigating the determinants of its development and onset holds great significance for preventive healthcare policy, health and social care practice, and the quality of life of individuals with dementia and their families.

Previous studies have found an association between wealth, as well as income, and the risk of dementia [7–10]. For example, a recent analysis based on representative longitudinal data from the English Longitudinal Study of Ageing showed an association between low wealth and a higher dementia risk [7]. Similarly, using longitudinal data from the Health and Retirement Study in the US, another investigation also found an association between lower wealth and income and a higher risk of dementia [10]. A further study showed that higher income was associated with lower odds of incident dementia based on data from the National Health and Aging Trends Study [11], whilst another study, using data from the Health, Aging, and Body Composition Study [12], also found an association between low income and increased hazard of dementia. Furthermore, a previous systematic review and meta-analysis showed an association between low income and combined risks of cognitive impairment and dementia [13]. Another recent systematic review and meta-analysis stressed the importance of a lower social class for the risk of developing dementia [14].

However, the limited number of existing studies, particularly those concentrating on the link between *wealth* and dementia risk [14], are predominantly focused on individuals in *old age* such as individuals aged 65 years and over. To date, there is limited knowledge on the link between wealth as well as income and dementia risk exclusively based on the *oldest old* (80 years and over).

Therefore, our aim was to examine the association between income, wealth and dementia based on representative, longitudinal data (including individuals living in institutionalized settings) of the oldest old. This knowledge has the potential to significantly enhance research on poverty and inequality. A link between income, wealth and dementia among the oldest old may be explained by lifestyle- or education-related factors [15, 16]. More precisely, factors such as occupation, diet, smoking and drinking habits, sport, lifelong learning, social and cognitive activities, access to healthcare and stress could explain the association between wealth and income and the risk of dementia [15, 17–19]. Further details regarding the potential mechanisms are provided in the discussion section. We expect that both higher income and wealth are associated with a lower likelihood of dementia.

Moreover, examining the association of wealth and income with dementia risk specifically among the oldest old is important as various critical life events take place in this age bracket, such as spousal loss, loss of friends and other relatives, admission to nursing home and serious health deteriorations (e.g., dual sensory impairment, immobility, frailty or cognitive decline) [20, 21]. Such critical life events reflect unique challenges. For example, an admission to a nursing home or the loss of the spouse may reflect financial challenges [22]. Additionally, health deteriorations such as sensory impairment may increase the likelihood of developing dementia [23].

## Methods

### Sample

This investigation utilized longitudinal data obtained from the “Survey on quality of life and subjective well-being of the very old in North Rhine-Westphalia (NRW80+)” (see [24]. The NRW80 + survey includes individuals aged 80 and older residing in North Rhine-Westphalia, which is the most populous state in Germany. It is worth noting that the socio-demographic distribution in North Rhine-Westphalia closely resembles that of the overall German population. The NRW80 + study covers a wide range of topics, including quality of life, assets, socioeconomic factors, social support, and health-related aspects.

A representative sample was drawn from nearly 100 communities in North Rhine-Westphalia. To ensure proper representation, men and individuals aged 85 years and above were oversampled. Therefore, sampling weights were utilized in the regression analysis to account for the complex survey design and account for attrition. The initial wave of data collection occurred between August 2017 and February 2018. For the second wave, data collection specifically targeted the panel sample (i.e., individuals who had already participated in the first wave) from June 2019 to February 2020. All variables included in this current study were quantified in both waves. On average, face-to-face interviews lasted approximately 80 to 90 min for participants (and about 60 to 70 min for proxy persons such as relatives or professional caregivers). Proxy interviews were used if the participant was not (any longer) able to participate in the interview himself/herself – mainly due to poor health status. For almost all of the proxy persons interviewed in the panel survey, it was stated that the target person had a care level or a degree of care.

In the first wave, the response rate was about 23%. Importantly, sociodemographic factors including age, living arrangement, and sex did not influence the likelihood of participation [24]. In the second wave, the response rate among the panel sample was nearly 57%. Our analytical sample consisted of 943 observations (details about missing data are shown in Supplementary File 1). Further details on the study have been described elsewhere [24].

Prior to participation, all participants or their legal representatives provided written informed consent. The NRW80 + study received ethical approval from the ethics committee of the Medical Faculty at the University of Cologne (No. 17–169). The NRW80 + study is in accordance with the Declaration of Helsinki and its later amendments.

### Study outcome: Dementia

To assess probable dementia, we utilized the DemTect screening tool [25, 26]. It has five subtests: Word list/delayed recall, number transcoding, verbal fluency, digit span reverse, and word list delayed recall. The five subtests took about 6–8 min to complete, including the instructions and evaluation of the raw scores. This tool can be carried out and evaluated objectively. To avoid overburdening physicians, this tool can also be carried out by appropriately trained staff. A detailed description of the subtests is given by Kalbe and Kessler [27].

Scores on the DemTect range from 0 to 18, with higher scores indicative of milder cognitive impairment. A score below 9 suggests the presence of dementia, while a score of 9 or above suggests the absence of dementia. DemTect has been found to be a reliable screening tool for dementia [25], with a specificity of 97% and a sensitivity of 85.1%.

### Independent variables: wealth and income

Our key independent variable were wealth and income, respectively. As for wealth, and in accordance with the acknowledged German Ageing Survey [28], individuals should consider all of their assets including savings accounts, investment contracts, life insurance, bonds, and valuable possessions owned by them or their spouse/partner, excluding real estate. Response categories were as follows: no assets; below 500 €; 500€ to below 2,500€; 2,500€ to 5,000€; 5,000€ to below 12,500€; 12,500€ to below 25,000€; 25,000€ to below 50,000€; 50,000€ to below 100,000€; 100,000€ to below 250,000€; 250,000€ to below 500,000€; 500,000€ or more. Subsequently, we developed four wealth quartiles: lowest wealth quartile: up to below 5,000€; second lowest wealth quartile: 5000€ to below 25,000€; second highest wealth quartile: 25,000€ to below 100,000€; highest wealth quartile: 100,000€ or more.

As for income, individuals reported their average monthly household net income (all sources of income minus taxes and social security contributions; for self-employed individuals: average net income, i.e., after deducting business expenses and taxes). Based on the calculated household equivalence net income, seven categories were given (below 1,000€; 1,000€ to below 1,500€; 1,500€ to below 2,000€; 2,000€ to below 3,000€; 3,000€ to below 5,000€; 5,000€ to below 18,000€; more than 18,000€). Based on these categories, we generated four income quartiles: lowest income quartile: up to below 1,500€; second lowest income quartile: 1,500€ to below 2,000€; second highest income quartile: 2,000€ to below 3,000€; highest income quartile: 3,000€ or more).

### Covariates

The selection of covariates in our analysis was guided by previous research in the field [14, 29] as well as theoretical considerations particularly related to the biopsychosocial model proposed by Spector and Orrell [30] and the life course model recently proposed by Fratiglioni et al. [31]. The biopsychosocial model includes both biopsychosocial factors that are fixed (e.g., age) and those that are modifiable (e.g., mental factors). According to the biopsychosocial model, age and education are clear risk factors for dementia. In addition, Spector and Orrell [30] emphasize the importance of pre-dementia health (e.g., certain chronic diseases) or mental health factors. More details are given by Spector and Orrell [30].

The life course model suggested by Fratiglioni et al. [31] highlights several risk factors of dementia including genetic, environmental, vascular, psychosocial, and lifestyle elements. They state that genetic and environmental factors, such as depression, synergistically interact in the development of dementia.

Thus, against the backdrop of data availability, we incorporated sociodemographic and health-related factors into our regression analysis as follows: age (in years), sex (men; women), education (classified according to ISCED-97 [32], as low, medium, and high education), and marital status (married, living together with spouse; Other: single, widowed, divorced, married, living separated from spouse).

Additionally, we incorporated the following health-related factors: self-rated health (single item from 1 = very bad to 4 = very good), depressive symptoms, and a count score for chronic conditions. Depressive symptoms were assessed using the “depression in old age scale” (DIA-S) [33, 34], which consists of four items (rated as either no or yes). A sum score was computed based on these four items, ranging from 0 to 4, with higher values indicating more depressive symptoms. The scale has demonstrated favorable psychometric properties [33, 34]. To measure chronic conditions, a count score was generated, incorporating 19 self-reported chronic illnesses. Each condition was scored as either 0 (absence) or 1 (presence). The chronic conditions included myocardial infarction, heart failure, hypertension, stroke, mental illness, cancer, diabetes, respiratory or pulmonary disease, back pain, gastric or intestinal disease, kidney disease, liver disease, blood disease, joint or bone disease, bladder disease, sleep disorder, eye disease or visual disorder, ear disease or hearing impairment, and neurological disease.

### Statistical analysis

We first described the sample characteristics to gain an initial understanding of our data. Thereafter, the bivariate association between income quartile and probable dementia, and the bivariate association between wealth quartile and probable dementia were calculated. Subsequently, a random effects (RE) logistic model was employed to estimate the association between wealth as well as income and probable dementia, adjusting for sex, age, marital status, educational level, self-rated health, depressive symptoms, and the number of chronic conditions. This panel econometric model, is widely utilized and recognized [35]. It utilizes both variations between and within individuals over time. Our analytical choice is also guided by the results of a Hausman-test (Chi²=6.48, *p* = 0.59), which helps to decide between RE- and FE models. Since the Hausman-test is non-significant, we prefer the RE-model because it is more efficient than the FE model. Apart from odds ratios (OR), we also report average marginal effects (AME) across the observed data (Stata command: “margins, dydx(*)” after the RE logistic regression). In other words: For each observation, the marginal effect is computed, and then the average of those effects is reported.

All statistical analyses were performed using Stata 16.1 (Stata Corp., College Station, Texas).

## Results

### Sample characteristics and bivariate associations

Table 1 shows characteristics of the study sample (for the analytical sample; pooled over both waves; baseline sample is shown in Supplementary File 1). The mean age was 86.0 years (SD: 4.0 years), with females accounting for about 44% of the participants. Most of the individuals had a medium level of education (52.7%). Table 1 provides additional detailed information.

**Table 1:** Characteristics of the analytical sample (unweighted, pooled over both waves)

	Total
	*N* = 943
Age: Mean (SD)	86.0 (4.0)
Sex: N (%)	
Men	525 (55.7%)
Women	418 (44.3%)
Marital status: N (%)	
Married, living separated from spouse; widowed; divorced; single	539 (57.2%)
Married, living together with spouse	404 (42.8%)
Educational level (ISCED-97): N (%)	
Low	158 (16.8%)
Medium	497 (52.7%)
High	288 (30.5%)
Self-rated health: N (%)	
Very bad	46 (4.9%)
Rather bad	265 (28.1%)
Rather good	542 (57.5%)
Very good	90 (9.5%)
Depressive symptoms: Mean (SD)	0.8 (1.0)
Count score: Chronic conditions: Mean (SD)	3.2 (2.1)
Wealth quartile: N (%)	
Lowest wealth quartile	225 (23.9%)
Second lowest wealth quartile	222 (23.5%)
Second highest wealth quartile	291 (30.9%)
Highest wealth quartile	205 (21.7%)
Income quartile: N (%)	
Lowest income quartile	313 (33.2%)
Second lowest income quartile	213 (22.6%)
Second highest income quartile	277 (29.4%)
Highest income quartile	140 (14.8%)
Probable dementia: N (%)	
Absence of probable dementia	890 (94.4%)
Presence of probable dementia	53 (5.6%)

Of note, the bivariate association between income quartile and probable dementia equaled Cramer’s V = 0.12 (Chi²=19.30, *p* < 0.001; *n* = 1,288). Moreover, the bivariate association between wealth quartile and probable dementia equaled Cramer’s V = 0.18 (Chi²=34.75, *p* < 0.001; *n* = 1,078). Furthermore, the bivariate association between income quartile and wealth quartile equaled Spearman’s rho = 0.46, *p* < 0.001 (*n* = 1,222; results are shown in Supplementary File 1).

### Regression analysis

Findings of the multiple logistic regression analysis are shown in Table 2 (additionally, marginal effects are shown in Table 3). The observations per individual varied from 1 to 2 (average: 1.6). The regression model adjusted for sex, age, marital status, educational level, self-rated health, depressive symptoms, and the number of chronic conditions. The regression analysis showed that greater wealth was independently associated with a lower likelihood of probable dementia. More precisely, compared to the lowest wealth quartile, the results were as follows for the second lowest wealth quartile: OR: 0.23, 95% CI: 0.07-0.76, AME: − 0.07; the second highest wealth quartile: OR: 0.05, 95% CI: 0.01-0.31, AME: − 0.11; and the highest wealth quartile: OR: 0.12, 95% CI: 0.02-0.91, AME: − 0.10. In contrast, in this model, income was not significantly associated with the likelihood of probable dementia.

**Table 2:** A logistic regression model showing the associations between wealth, income and probable dementia (*N* = 943)

Independent variables	OR (95% CI)	OR (95% CI) – model without wealth
Wealth quartile: - Second lowest wealth quartile (Reference category: Lowest wealth quartile)	0.23*	
	(0.07–0.76)	
- Second highest wealth quartile	0.05**	
	(0.01–0.31)	
- Highest wealth quartile	0.12*	
	(0.02–0.91)	
Income quartile: - Second lowest income quartile (Reference category: Lowest Income quartile)	0.67	0.49
	(0.22–2.03)	(0.19–1.26)
- Second highest income quartile	0.67	0.27*
	(0.19–2.39)	(0.09–0.86)
- Highest income quartile	0.16	0.12+
	(0.01–3.31)	(0.01–1.04)
Covariates^a^	✓	✓
Observations	943	1,164
Number of Individuals	596	699

The reported results include Odds Ratios, presented with corresponding 95% confidence intervals (CI); weights were used

*** *p* < 0.001, ** *p* < 0.01, * *p* < 0.05, + *p* < 0.10

^a^Covariates include: sex, age, marital status, educational level, self-rated health, depressive symptoms and the number of chronic conditions

**Table 3:** A logistic regression model showing the associations between wealth, income and probable dementia (*N* = 943) – showing average marginal effects

Independent variables	AME (95% CI)	AME (95% CI) – model without wealth
Wealth quartile: - Second lowest wealth quartile (Reference category: Lowest wealth quartile)	−0.07**	
	(−0.13 - −0.02)	
- Second highest wealth quartile	−0.11***	
	(−0.17 - −0.06)	
- Highest wealth quartile	−0.10**	
	(−0.16 - −0.03)	
Income quartile: - Second lowest income quartile (Reference category: Lowest Income quartile)	−0.02	−0.03
	(−0.06–0.03)	(−0.08–0.01)
- Second highest income quartile	−0.02	−0.06*
	(−0.07–0.03)	(−0.10 - −0.01)
- Highest income quartile	−0.06+	−0.08**
	(−0.12–0.01)	(−0.13 - −0.02)
Covariates^a^	✓	✓
Observations	943	1,164
Number of Individuals	596	699

The reported results include average marginal effects (AME), presented with corresponding 95% confidence intervals (CI); weights were used

*** *p* < 0.001, ** *p* < 0.01, * *p* < 0.05, + *p* < 0.10

^a^Covariates include: sex, age, marital status, educational level, self-rated health, depressive symptoms and the number of chronic conditions

When we removed wealth from our main model (please see Tables 2 and 3), however, income quartiles partly achieved statistical significance. More precisely, compared to the lowest income quartile, the results were as follows for the second lowest income quartile: OR: 0.49, 95% CI: 0.19-1.26, AME: − 0.03; the second highest income quartile: OR: 0.27, 95% CI: 0.09-0.86, AME: − 0.06; and the highest income quartile: OR: 0.12, 95% CI: 0.01-1.04, AME: − 0.08.

## Discussion

Our objective was to investigate the association between income, wealth and probable dementia. Our results showed a significant association between greater wealth and a lower likelihood of probable dementia, whereas no significant association was identified between income and probable dementia in our main analysis. Former studies, for example, mainly focused on individuals in *old age* and consistently showed a link between low wealth and a higher risk of dementia based on representative samples from the United States or England [7–10]. Overall, our study expands the very limited knowledge base around these variable as it uniquely focuses on the oldest old population, and utilizes longitudinal data obtained from a representative sample, which includes individuals living in institutionalized settings as well. The notable association of wealth and dementia risk identified in our current study might be attributed to the widely recognized concept of cognitive reserve or brain reserve capacity theory [16] – potentially based on education-related factors. The cognitive reserve theory mainly refers to the capacity of the brain to sustain optimal cognitive functioning even in the presence of brain pathology or damage. It posits that individuals with a greater cognitive reserve are more adept at enduring and compensating for age-related cognitive decline or neurological impairments compared to those with a lower cognitive reserve [36]. The brain reserve capacity theory states that individuals with greater brain volume or structural complexity are better able to withstand brain damage before noticeable cognitive impairment occurs. This theory suggests that individuals with a higher brain reserve, e.g., with larger brain volumes or more neural connections, may be more resilient to the effects of ageing, neurological diseases or other cognitive challenges [37]. Wealth may facilitate access to lifelong education and may enrich experiences [19], which in turn may contribute to a more robust cognitive reserve and ultimately to a lower risk of developing dementia [38]. Additionally, aligning with the cognitive reserve theory, the link between wealth (which is associated with high education [39]) and probable dementia may be attributed to lifestyle factors, such as nutrition or an active lifestyle [15] or access to better healthcare (including preventative measures). It is plausible that wealth is associated with human behavior and lifestyle practices [15], which in turn could contribute to a decreased likelihood of probable dementia in advanced age [40]. Moreover, low wealth may be associated with higher levels of stress [19]. Chronic stress can have a negative impact on brain health [17, 18]. Röhr et al. [41] also suggested the following as top priorities for the future: “creating good sustainable socioeconomic living standards for everyone” (p. 1531). 

Prior research also suggests an association between wealth and digital literacy, which [8] in turn is linked to a lower risk of dementia. In addition, as has been seen in LMICs, people with greater wealth tend to have a stronger constellation of social convoy encompassing many social relationships and positive interactions with others. Effective social connection has also been proven to delay mental disorders and cognitive dysfunction, including dementia [42]. Of note, our findings regarding wealth and probable dementia extend our current knowledge mainly based on older adults [7–10].

Interestingly, when including wealth in our regression model, income was not significantly associated with the risk of probable dementia. However, when wealth was removed from our main model, income gained statistical significance. This stresses the relevance of assets in general and the relevance of the model specification when examining the link between financial factors (such as wealth and income) and dementia risk. In addition, the money available in retirement is increasingly dependent on accumulated assets [43]. A key distinction between income and wealth is that income reflects the flow of financial resources over a specific timeframe, whereas wealth represents a cumulative stock shaped by a lifetime of decisions and influences [44]. Similarly, cognition acts as a cumulative measure, reflecting the impact of previous experiences [44]. Therefore, due to their cumulative characteristics, it is reasonable to expect that the relationship between wealth and cognition is present in later life. Moreover, statistical power may also be an issue.

The non-significant findings actually align with a recent meta-analysis which could not identify a significant link between income and dementia risk [14]. Interestingly, this former meta-analysis also showed that the operationalization of income varied quite markedly between the studies. For example, some studies used a fixed amount of income, whereas others used a percentage of the poverty rate [14]. Moreover, former research identifying a link between higher income and a lower risk of dementia commonly did not adjust for wealth [45]. In view of this, we recommend future research exploring the link between different sources of income (e.g., higher pension due to high earnings in working age; higher social benefits) in relation to dementia risk among the oldest old.

Some important strengths and limitations are worth noting: This study utilized a *longitudinal* dataset based on individuals aged *80 and above*, residing in both *private households and institutionalized settings*. Although the response rate was relatively low (e.g., it may be more likely that more vulnerable groups did not take part), it is important to recognize that this study provides a representative sample of the population aged 80 in North Rhine-Westphalia (Germany) [24]. Furthermore, sampling weights were employed in this study (e.g., to address attrition). Established tools were utilized to quantify the independent variables, and a screening tool was employed to assess probable dementia. Future research based on clinical diagnoses of dementia are recommended to corroborate our findings. Like in previous studies, the measurement of self-assessed overall wealth and income was employed. As far as data are available, objective information on asset values (and income) would be desirable for future studies since previous research has found that the probability of overreporting is significantly higher at the lower end of the wage distribution and, conversely, that the probability of underreporting is higher at the upper end [46]. In this respect, the true effects could probably be underestimated in this study; it should also be mentioned here that particularly vulnerable groups are likely to participate less frequently in research. Moreover, the follow-up period was relatively short in our current study. Additionally, further covariates may be desirable (e.g., early life socioeconomic status). Furthermore, the possibility of reverse causality cannot be dismissed (e.g., poor cognitive function can impair the ability to manage money, further reducing income and wealth levels).

In conclusion, our study showed a link between wealth and dementia. This current study adds significantly to the currently scarce understanding of the oldest old by specifically examining this age group and using longitudinal data from a representative sample that includes individuals residing in institutionalized settings.

Such knowledge can enrich the poverty and inequality research within this field. More specifically, our results provide backing for policy interventions grounded in empirical evidence, which seek to alleviate the impact of dementia potentially by addressing the reduction of economic disparities or by improving the distribution of income in later life and across the life course. Additionally, general practitioners could be more aware of dementia and its prevention for individuals with limited financial resources. This can facilitate early intervention and support measures tailored to those individuals. Furthermore, interventions designed to aid older individuals facing financial challenges to meet their basic needs may be helpful. Moreover, developing sustainable socioeconomic living standards for all may be crucial.

Future research should examine the influences of financial resources over the life course on the development of dementia in later life. Moreover, further investigations are warranted that shed light on the potential underlying mechanisms through which economic factors influence dementia risk in older adults.

## Supplementary Information

Supplementary Material 1.

## Data Availability

The NRW 80+ data are available via gesis. For interested researchers, please see: https://search.gesis.org/research_data/ZA7558.
