# Supplementary material for: Wealth, income and dementia in Germany: longitudinal findings from a representative survey among the oldest old
Source: BMC Public Health. 2025 Aug 29;25:2965. doi: 10.1186/s12889-025-24239-1 (PMC12395681; doi:10.1186/s12889-025-24239-1)
Supplement: Supplementary file 1 — Supplementary Material 1. [file 12889_2025_24239_MOESM1_ESM.docx]

Number and proportion of missing values

Variable | Missing Total Percent Missing

-------------------+-----------------------------------------------

Dementia | 346 1,824 18.97

Wealth quartile | 564 1,824 30.92

Income quartile | 282 1,824 15.46

Age | 8 1,824 0.44

Marital status | 0 1,824 0.00

Education | 108 1,824 5.92

Sex | 0 1,824 0.00

Self-rated health | 5 1,824 0.27

Depressive sympt. | 123 1,824 6.74

Chronic conditions | 19 1,824 1.04

Characteristics of the analytical sample (unweighted, baseline)

|  | Total |
| --- | --- |
|  | N=473 |
| Age: Mean (SD) | 85.1 (3.9) |
| Sex: N (%) |  |
| Men | 259 (54.8%) |
| Women | 214 (45.2%) |
| Marital status: N (%) |  |
| Married, living separated from spouse; widowed; divorced; single | 263 (55.6%) |
| Married, living together with spouse | 210 (44.4%) |
| Educational level (ISCED-97): N (%) |  |
| Low | 83 (17.5%) |
| Medium | 249 (52.6%) |
| High | 141 (29.8%) |
| Self-rated health: N (%) |  |
| Very bad | 21 (4.4%) |
| Rather bad | 130 (27.5%) |
| Rather good | 272 (57.5%) |
| Very good | 50 (10.6%) |
| Depressive symptoms: Mean (SD) | 0.7 (1.0) |
| Count score: Chronic conditions: Mean (SD) | 3.3 (2.1) |
| Wealth quartile: N (%) |  |
| Lowest wealth quartile | 124 (26.2%) |
| Second lowest wealth quartile | 104 (22.0%) |
| Second highest wealth quartile | 142 (30.0%) |
| Highest wealth quartile | 103 (21.8%) |
| Income quartile: N (%) |  |
| Lowest income quartile | 171 (36.2%) |
| Second lowest income quartile | 101 (21.4%) |
| Second highest income quartile | 132 (27.9%) |
| Highest income quartile | 69 (14.6%) |
| Probable dementia: N (%) |  |
| Absence of probable dementia | 450 (95.1%) |
| Presence of probable dementia | 23 (4.9%) |

Bivariate association between income quartiles and dementia.

| 4 quartiles of income

Dementia | 1 2 3 4 | Total

-----------+--------------------------------------------+----------

Absence | 411 272 336 181 | 1,200

| 90.33 90.97 96.55 97.31 | 93.17

-----------+--------------------------------------------+----------

Presence | 44 27 12 5 | 88

| 9.67 9.03 3.45 2.69 | 6.83

-----------+--------------------------------------------+----------

Total | 455 299 348 186 | 1,288

| 100.00 100.00 100.00 100.00 | 100.00

Pearson chi2(3) = 19.3049 Pr = 0.000

Likelihood-ratio chi2(3) = 21.2074 Pr = 0.000

Cramér's V = 0.1224

gamma = -0.3517 ASE = 0.076

Kendall's tau-b = -0.1048 ASE = 0.023

Bivariate association between wealth quartiles and dementia.

| 4 quartiles of wealth

Dementia | 1 2 3 4 | Total

-----------+--------------------------------------------+----------

Absence | 246 236 307 215 | 1,004

| 86.01 93.28 97.46 95.98 | 93.14

-----------+--------------------------------------------+----------

Presence | 40 17 8 9 | 74

| 13.99 6.72 2.54 4.02 | 6.86

-----------+--------------------------------------------+----------

Total | 286 253 315 224 | 1,078

| 100.00 100.00 100.00 100.00 | 100.00

Pearson chi2(3) = 34.7503 Pr = 0.000

Likelihood-ratio chi2(3) = 33.0726 Pr = 0.000

Cramér's V = 0.1795

gamma = -0.4598 ASE = 0.085

Kendall's tau-b = -0.1436 ASE = 0.028

Spearman correlation: Income quartile and wealth quartile

Number of observations = 1,222

Spearman's rho = 0.4593
